# Hearing Outcomes During Induction Therapy in ANCA-Associated Vasculitis: Applicability of Sudden Sensorineural Hearing Loss Criteria

**DOI:** 10.3390/jcm15114349

**Published:** 2026-06-04

**Authors:** Michał Stanisław Kaczmarczyk, Sandra Krzywdzińska, Paweł Rozbicki, Jacek Usowski, Marcin Jadczak, Dariusz Jurkiewicz, Maria Sobol, Stanisław Niemczyk, Elżbieta Głuch, Ksymena Leśniak

**Affiliations:** 1 Department of Otolaryngology and Laryngological Oncology with Clinical Department of Craniofacial Surgery, Military Institute of Medicine—National Research Institute, Szaserów 128, 04-349 Warsaw, Poland; 2 Department of Biophysics, Physiology and Pathophysiology, Medical University of Warsaw, 02-901 Warsaw, Poland; maria.sobol@wum.edu.pl; 3 Department of Internal Diseases, Nephrology and Dialysis, Military Institute of Medicine—National Research Institute, Szaserów 128, 04-349 Warsaw, Poland

**Keywords:** ANCA, SSNHL, hearing loss, vasculitis, AAV

## Abstract

**Objectives**: ANCA-associated vasculitis (AAV) is a relapsing–remitting systemic disease in which auditory involvement is increasingly recognized as a determinant of quality of life. The objective of this study was to evaluate hearing outcomes during induction therapy in AAV and to assess the applicability of sudden sensorineural hearing loss (SSNHL) criteria for monitoring treatment response. **Methods**: A prospective study included 27 patients with AAV hospitalized at the Department of Internal Medicine, Nephrology and Dialysis, Military Institute of Medicine–National Research Institute. Audiologic assessment included pure-tone audiometry (including high-frequency audiometry), immittance audiometry, otoacoustic emissions, and auditory brainstem response (ABR). Examinations were performed at baseline (induction phase) and after remission. Hearing outcomes were evaluated using SSNHL criteria defined by the Ministry of Health and Welfare of Japan. Associations between hearing loss and organ involvement were also analyzed. **Results**: Improvement to normal hearing in pure-tone audiometry was observed in three ears, while deterioration from normal hearing occurred in four ears. No statistically significant differences were found in overall otoacoustic emission responses between baseline and follow-up. A statistically significant association was identified between hearing loss and peripheral nervous system involvement (50.0% vs. 11.1%, *p* = 0.057). No significant correlations were observed between hearing outcomes and other clinical parameters. **Conclusions**: SSNHL-based hearing outcome criteria may represent a practical tool for assessing auditory dysfunction and monitoring treatment response in AAV. High-frequency audiometry and structured outcome measures may improve detection of subtle cochlear changes. Further studies with larger cohorts are required to validate these findings and clarify the relationship between systemic disease activity and hearing impairment.

## 1. Introduction

ANCA-associated vasculitides (AAVs) are multisystem disorders of unclear etiology. Although the kidneys, lungs, and paranasal sinuses represent the most commonly affected organs, the auditory system should not be overlooked, given the essential role of hearing in social functioning and quality of life. Auditory involvement has been reported in up to 40% of patients with AAV [1,2], with sensorineural hearing loss—typically of cochlear origin—being the predominant type.

Two pathogenetic mechanisms have been proposed. The first involves an acute onset of sensorineural hearing loss resembling idiopathic sudden sensorineural hearing loss (SSNHL). The second describes a progressive course beginning with conductive hearing loss, evolving into mixed, and ultimately sensorineural hearing loss [3]. Regardless of the underlying mechanism, early diagnosis and prompt initiation of appropriate therapy are critical, as they may result in hearing recovery or at least limit irreversible damage associated with disease activity.

AAV remains an incurable condition characterized by a relapsing–remitting course, necessitating continuous clinical monitoring and patient awareness of early warning symptoms. According to current guidelines from the European Alliance of Associations for Rheumatology (EULAR) and the American College of Rheumatology (ACR), induction therapy consists of high-dose glucocorticoids combined with either cyclophosphamide or rituximab, which demonstrate comparable efficacy but differ in safety profiles. Alternative strategies include plasma exchange for rapidly progressive glomerulonephritis and avacopan for patients with glucocorticoid intolerance in granulomatosis with polyangiitis (GPA) and microscopic polyangiitis (MPA) [4].

In the present study, we evaluated changes in audiologic parameters during induction therapy in patients with AAV. Hearing outcomes were assessed using criteria established by the Research Committee of the Ministry of Health and Welfare in Japan for SSNHL [5,6]. Additionally, we analyzed changes in high-frequency audiometry, impedance audiometry, and otoacoustic emissions following treatment. Early identification of auditory deterioration may facilitate diagnosis of the AAV and timely initiation of induction therapy, potentially allowing for reversible hearing impairment.

## 2. Patients and Methods

### 2.1. Patients

The study cohort consisted of patients with proteinase 3 (PR3)-ANCA and myeloperoxidase (MPO)-ANCA hospitalized at the Department of Internal Medicine, Nephrology and Dialysis, Military Institute of Medicine–National Research Institute between December 2023 and February 2025. All patients were evaluated during the active phase of inflammation, either at first or subsequent induction therapy, regardless of self-reported hearing impairment. The first visit was carried out at the time of qualification for AAV remission-inducing treatment, and the second visit after the completion of this treatment. Informed consent was obtained from all individual participants included in the study.

Diagnosis of GPA and MPA was established according to the 2022 ACR/EULAR classification criteria [7,8]. Two rituximab (RTX) induction regimens were used: the RAVE protocol (375 mg/m^2^ weekly for four weeks), approved in the European Union for induction of remission in GPA and MPA [9], and a two-dose regimen (1 g at weeks 0 and 2), originally approved for rheumatoid arthritis [10]. In cases of severe renal involvement, a combination of RTX infusions and two pulses of intravenous cyclophosphamide was administered [11]. For the purpose of analysis, all RTX-based regimens were categorized as a single RTX treatment group. Intravenous pulse cyclophosphamide was administered according to the CYCLOPS protocol [12].

Exclusion criteria included congenital or early-onset deafness unrelated to vasculitic ear involvement (excluded affected ear), exposure to ototoxic medications (gentamicin, amikacin, tobramycin, high-dose furosemide, erythromycin, high-dose aspirin, cisplatin, or quinine), prior head and neck radiotherapy, meningitis, hereditary hearing disorders, and previous ear surgery unrelated to vasculitis (excluded operated ear).

All patients completed standardized questionnaires to obtain baseline clinical data. Disease activity was assessed using the Birmingham Vasculitis Activity Score for Wegener’s Granulomatosis (BVAS/WG). Additional variables included disease duration and number of relapses [13].

### 2.2. Audiologic Assessment

All participants underwent comprehensive audiologic evaluation at baseline (initiation of induction therapy) and after approximately six months, corresponding to the expected remission phase. The test battery included pure-tone audiometry (standard and high-frequency ranges), immittance audiometry, otoacoustic emissions (OAEs), and auditory brainstem response (ABR).

Pure-tone audiometry was performed using an Interacoustics AC40 audiometer (Interacoustics headquarters, Audiometer Allé 1, Middelfart, Denmark) with Sennheiser HDA 300 headphones. Air conduction thresholds were measured across standard frequencies (125–8000 Hertz (Hz)) and extended high frequencies (9000–16,000 Hz), while bone conduction thresholds were assessed within the conventional range (125–4000 Hz). Masking was applied when appropriate. Hearing loss was defined as a pure-tone average (PTA) > 20 decibels (dB). The conventional PTA Air Conduction/Bone Conduction (PTA AC/BC) was calculated as the mean threshold at 500, 1000, 2000, and 4000 Hz and classified according to World Health Organization criteria [14]. Additionally, a High-Frequency PTA (HF-PTA) was calculated as the mean threshold between 9000 and 12,500 Hz [15].

Hearing severity and treatment-related improvement were evaluated according to the Japanese Ministry of Health criteria for sudden sensorineural hearing loss. Hearing deterioration was graded based on PTA values (<40 dB, 40–59 dB, 60–89 dB, ≥90 dB). Improvement was categorized as no (<10 dB), slight (10–29 dB), marked (≥30 dB), or complete (final PTA < 20 dB or recovery to the contralateral ear level). Frequency-specific threshold changes were also analyzed.

Immittance audiometry (Interacoustics Titan IMP440) included tympanometry (226 Hz probe tone) and acoustic reflex testing at 500, 1000, and 2000 Hz. Tympanograms were classified as type A/Ad/As (considered functionally normal) or type B/C (abnormal). Improvement was defined as normalization from type B or C to A/Ad/As.

OAEs were recorded using the Otometrics Madsen Capella system (Natus Sensory Inc. 2800 W. Higgins Road, Suite 1100, Hoffman Estates, IL 60169, USA) to assess outer hair cell function. Transient-evoked (TEOAE) and distortion-product (DPOAE) responses were obtained using standard clinical parameters, Signal to Noise Ratio (SNR) ≥ 6 dB. DPOAEs were measured between 500 and 8000 Hz, and TEOAEs within frequency bands spanning 750–4500 Hz. Improvement was defined as the emergence of reproducible responses within previously absent frequency bands.

ABR recordings (Racia-Alvar system with Centor C software (Racia-Alvar CTO971205118, Centor-O software ver4.15 PL, Le Bouscat, France)) were performed using surface electrodes placed at the vertex and bilateral mastoids, with a ground electrode on the forehead. Click stimuli of alternating polarity were delivered monaurally at 90 dB SPL, with a stimulus rate of 20 stimuli per second. Each recording consisted of at least 1500 sweeps within a 12.5 ms analysis window. Responses were obtained ipsilaterally.

Absolute wave latencies (I, III, V), interpeak intervals (I–III, III–V, I–V), interaural latency differences in wave V (normal ≤0.4 ms), and the wave V/I amplitude ratio (normal >0.5) were analyzed. Wave morphology and reproducibility were also assessed to ensure recording reliability. ABR findings were used to support the diagnosis and localization of sensorineural hearing loss but were not included in statistical analyses.

### 2.3. Statistical Analysis

Data were analyzed using Statistica software (version 13.3; TIBCO Software Inc., Palo Alto, CA, USA). Quantitative variables were summarized using descriptive statistics, including the mean, standard deviation (SD), median, and range. Categorical variables were expressed as frequencies and percentages. Associations between categorical variables were assessed using the chi-square test or Fisher’s exact test, as appropriate. The normality of quantitative variable distributions was evaluated using the Shapiro–Wilk test. As the assumption of normality was not met, comparisons between pre- and post-treatment measurements were performed using the Wilcoxon signed-rank test. A significance level of α = 0.05 was applied.

## 3. Results

### 3.1. General Characteristics

The study included 27 patients, comprising 16 men (59.3%) and 11 women (40.7%), hospitalized at the Department of Internal Diseases, Nephrology and Dialysis, Military Institute of Medicine—National Research Institute between December 2023 and September 2025. The mean age was 54.7 ± 14.8 years (median 56 years, range 20–77 years). The mean disease duration was 16.5 ± 32.5 months (median 2 months; range 0–163 months). Among the patients, 20 (74.1%) experienced their first manifestation of the disease, 5 (18.5%) their second, and 2 (7.4%) their third. The mean BVAS-WG score was 9.4 ± 4.2 (median 9, range 1–20). Sixteen (59.3%) of the 27 patients were treated with RTX, while 11 (40.7%) received CF. Among the 27 patients, 16 were positive for PR3 ANCA, nine for MPO ANCA, and two were double negative. The mean interval between first and second visit was 6.72 ± 1.76 months (range 3–8 months).

### 3.2. Audiologic Tests

#### 3.2.1. Pure Tone Audiometry

**Figure 1:**
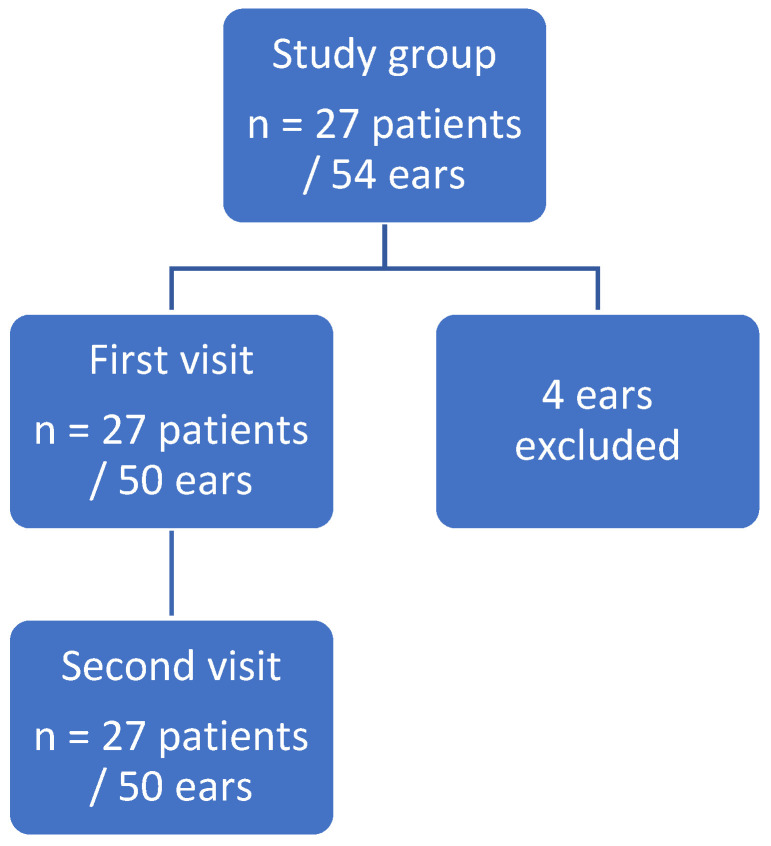
Patient flow diagram.

Audiometric data were obtained from 50 ears of 27 patients in the study group. Four ears were excluded from analysis due to predefined exclusion criteria, while the corresponding patients remained in the study (three ears with surgery anamnesis prior to AAV diagnosis, one with early congenital hearing loss) (Figure 1). At the first visit, pure-tone audiometry identified hearing loss in 28 ears. Of these, 12 ears demonstrated sensorineural hearing loss, 9 showed conductive hearing loss, and 7 exhibited mixed hearing loss. At the second visit, pure-tone audiometry identified hearing loss in 26 ears. Among them, 11 ears demonstrated sensorineural hearing loss, 8 showed conductive hearing loss, and 7 exhibited mixed hearing loss. No statistically significant differences were observed between the two visits.

During analysis of the improvement in specific frequencies in pure tone audiometry, statistically significant differences were observed between the first (pre-treatment) and second (post-treatment) assessments. These differences were found only at the frequency of 6000 Hz in air conduction (AC) measurements (*p* = 0.019) and at 10,000 Hz in high-frequency audiometry (*p* = 0.033). At 6000 Hz, the post-treatment sound threshold was significantly lower compared to the pre-treatment level whereas at 10,000 Hz, the post-treatment threshold was significantly higher than before treatment.

Furthermore, hearing improvement was evaluated based on the pure-tone average (PTA) and high-frequency pure-tone average (PTA-HFA; 9000–12,500 Hz). In the speech frequency range (500–4000 Hz), a reduction in hearing threshold of more than 10 dB but less than 30 dB was observed in 4 of 50 examined ears. In the high-frequency range (PTA-HFA), a similar improvement was noted in 6 of 35 ears.

Improvement to normal hearing occurred in three ears (two from conductive and one from sensorineural hearing loss), while deterioration from normal hearing was observed in four ears (three to sensorineural and one to conductive hearing loss). Changes in hearing loss classification were also common: two ears converted from sensorineural to conductive hearing loss and four to mixed hearing loss. Moreover, among ears with initial conductive hearing loss, three transitioned to sensorineural hearing loss and one to mixed hearing loss then among ears with mixed hearing loss, three converted to sensorineural hearing loss and two to conductive hearing loss.

Organ involvement was compared between patients with normal hearing and those with hearing loss. No statistically significant differences were observed between the groups for ocular, central nervous system, joint, gastrointestinal, cardiac, renal, cutaneous, sinonasal, or pulmonary involvement (all *p* > 0.05). Peripheral nervous system involvement showed the greatest difference between groups, although the result did not reach statistical significance (*p* = 0.057). It was present in 50.0% of patients with hearing loss compared with 11.1% of patients with normal hearing (Table 1).

Based on the Hearing Improvement Criteria for Sudden Sensorineural Hearing Loss (SSHL) defined by the Ministry of Health and Welfare of Japan, patients were categorized according to a proposed grading system both before and after treatment. Hearing loss was classified into four grades (1–4) [1,2]. As it was written before, hearing recovery outcomes were further categorized into four groups: complete recovery, marked improvement, slight improvement, and no change.

No statistically significant difference was observed between pre- and post-treatment visits with respect to changes in hearing loss grade or hearing loss type (Table 2 and Table 3). Improvement from grade 3 (G3) to grade 2 (G2) was observed in one ear, and from grade 2 (G2) to grade 1 (G1) in four ears; however, these changes were not statistically significant.

Due to the limited number of patients within individual subgroups, formal statistical comparisons were not performed, and the following findings should be interpreted as descriptive observations only. Among patients with MPO-ANCA positivity (17 ears), slight improvement in standard PTA frequencies was observed in 5.9% of ears, while 17.6% of ears demonstrated improvement at extended high frequencies. In the PR3-ANCA group (29 ears), slight improvement was observed in 3.4% of ears in standard PTA frequencies and at 10.3% of ears at extended high frequencies. In double-negative patients (4 ears), 50% of ears showed slight improvement in standard PTA frequencies, although no improvement was observed at extended high frequencies.

Regarding treatment type, slight improvement in standard PTA frequencies was observed in 9.7% of ears treated with RTX (31 ears) and in 5.3% of ears treated with CF. Improvement at extended high frequencies was observed in 19.2% of RTX-treated ears and in 10.5% of CF-treated ears (19 ears).

In patients with disease duration shorter than 2 months (24 ears), slight improvement in standard PTA frequencies was observed in 8.3% of ears, while 12.5% of ears showed improvement at extended high frequencies. No cases of marked improvement or complete recovery were observed in the groups analyzed.

#### 3.2.2. Otoacoustic Emission and ABR Analysis

In the case of otoacoustic emissions, new responses were detected across multiple frequency bands. The number of newly observed responses was as follows: seven at DPOAE-gram print 1 kHz, six at TEOAE 1.75–2.5 kHz, five at SOE and DPOAE-gram print 0.5 kHz, 1.5 kHz, 3 kHz, 4 kHz, and 6 kHz, as well as TEOAE 0.75–1.25 kHz; four at TEOAE 2.5–3.5 kHz; three at DPOAE-gram print 0.75 kHz and 8 kHz; and two at TEOAE 3.5–4.5 kHz. Despite the improvement in specific frequency bands, there were no statistically significant differences between overall OEA responses between the first and second visits (Table 4).

Mean wave V latency at the first visit was 6.0 ± 0.4 ms (median 6.0 ms; range 5.4–7.1 ms), compared with 5.9 ± 0.4 ms (median 5.9 ms; range 5.2–7.2 ms) at follow-up. These findings were within expected normative ranges and did not suggest retrocochlear conduction abnormalities, supporting a predominantly cochlear origin of hearing loss in our cohort.

#### 3.2.3. Analysis of Subgroups Stratified by Disease Duration

Given that chronic middle ear disease is conventionally defined using a temporal threshold of approximately 3 months, we performed an exploratory subgroup analysis to evaluate whether earlier versus longer disease duration might influence audiologic findings. For this purpose, the cohort was divided according to the time from symptom onset to diagnosis: Group 1 included patients with disease duration longer than 2 months, while Group 2 included those with disease duration shorter than 2 months. This cutoff was selected pragmatically to distinguish potentially reversible early otologic manifestations from more established disease.

In impedance audiometry, an improvement in tympanometric curve type was observed in 2 of 50 examined ears. In one case, the curve changed from type C to type Ad, and in the other from type C to type A. In the first case, improvement in otoacoustic emissions was observed only at 4 kHz, whereas in the second, improvement was noted at 1 kHz.

Statistically significant differences were observed in the distribution of tympanometric curve types between these subgroups. At both pre- and post-treatment assessments, type A tympanograms were more frequent in Group 1 than in Group 2 (pre-treatment: 17 ears [70.8%] vs. 9 ears [42.1%]; post-treatment: 18 ears [75.0%] vs. 9 ears [45.0%], respectively). No patients demonstrated a type C tympanogram at follow-up.

No statistically significant differences were observed between disease-duration subgroups in the distribution of overall DPOAE, TEOAE, or hearing loss categories at either assessment (Table 5).

## 4. Discussion

Monitoring hearing outcomes after systemic therapy is a critical component of audiologic care, particularly in disorders with inflammatory or immune-mediated pathophysiology. Among otologic entities, sudden sensorineural hearing loss remains the most extensively investigated condition with respect to prognosis and treatment response. With an annual incidence of 5–20 cases per 100,000 individuals, SSNHL has provided a framework for defining recovery criteria and therapeutic endpoints in cochlear disorders [16]. Although its exact pathophysiology remains incompletely elucidated, vascular compromise, immune-mediated injury, and viral-triggered inflammation have all been implicated. These mechanisms overlap conceptually with those proposed in ANCA-associated vasculitis (AAV), making SSNHL criteria a reasonable reference model for outcome assessment in vasculitis-related hearing loss.

Auditory involvement in AAV has gained increasing attention as overall survival improves and quality-of-life outcomes become more clinically relevant. Histopathologic and imaging studies suggest that cochlear dysfunction in AAV is predominantly related to inflammation of the stria vascularis and small-vessel vasculitis within the cochlear microcirculation [3]. The stria vascularis plays a central role in maintaining the endocochlear potential and ionic homeostasis; therefore, even subtle inflammatory or ischemic injury may disrupt outer hair cell function and high-frequency hearing before affecting conventional PTA thresholds. This microvascular hypothesis provides a biologically plausible explanation for the early vulnerability of basal cochlear regions in AAV. Notably, conductive hearing loss was more frequently observed in patients with shorter disease duration, whereas sensorineural hearing loss predominated in patients with longer-standing disease, suggesting that middle ear involvement may be particularly relevant in earlier disease stages, while cochlear dysfunction may become more prominent over time (Table 5). This pattern may reflect the underlying pathophysiology. In immune-mediated cochlear injury, structural damage to outer hair cells or spiral ganglion neurons may become irreversible once a certain threshold of ischemic or inflammatory insult is exceeded. Therefore, partial improvement may represent restoration of microvascular perfusion and resolution of inflammatory edema rather than true regeneration of sensory structures.

To standardize outcome assessment, we applied the Japanese Ministry of Health grading system for SSNHL [5,17], aiming to determine its applicability in AAV-related hearing impairment. In our cohort of 27 patients (50 ears), sensorineural hearing loss was the predominant subtype (24%). Only four ears fulfilled criteria for slight improvement. Although these changes did not reach statistical significance, the observed pattern suggests that recovery, when present, tends to occur in ears with moderate baseline impairment and in those with mixed or conductive components. The most frequent shift in hearing grade was from Grade III to Grade II (Table 2), indicating partial rather than complete cochlear recovery. An important aspect of the present study was not only the assessment of hearing changes during induction therapy, but also the evaluation of whether an established outcome classification could be pragmatically adapted to AAV-related hearing impairment. Unlike idiopathic SSNHL, hearing loss in AAV may be bilateral, fluctuate over time, or occur in patients with pre-existing hearing deficits, making some conventional recovery classifications less directly applicable. In this context, the Japanese Ministry of Health and Welfare SSNHL criteria offered several practical advantages, including independence from contralateral ear comparison, no requirement for knowledge of premorbid hearing status, and reliance on pure-tone audiometry as the principal outcome measure. Although originally developed for idiopathic SSNHL, this framework allowed structured grading of both baseline hearing severity and treatment-related improvement in our cohort. Given the absence of disease-specific audiologic outcome classifications for AAV, such standardized approaches may improve comparability between future studies.

High-frequency audiometry (HFA) provides additional insight into this process. High-frequency thresholds are particularly sensitive to early cochlear compromise due to the tonotopic arrangement of the organ of Corti, where basal regions encoding higher frequencies are metabolically active and structurally exposed. In a Japanese nationwide SSNHL survey, complete or marked recovery occurred in 30% of cases, partial recovery in 30%, and no recovery in 40%, with profound baseline deafness associated with irreversible loss [18]. In our cohort, slight improvement in HFA was observed in six of 35 ears.

Importantly, in our recent study we demonstrated that patients with AAV and normal conventional PTA (500–4000 Hz) already exhibit significantly elevated thresholds above 8000 Hz compared with controls without AAV [19]. This finding supports the concept that high-frequency dysfunction may represent a subclinical marker of early cochlear vasculitis. Consequently, even modest improvement in HFA may indicate restoration of cochlear microcirculation and stabilization of inflammatory activity. From a translational perspective, HFA may therefore serve as a functional biomarker of reversible microvascular injury.

Frequency-specific analysis revealed statistically significant improvement at 6000 Hz and deterioration at 10,000 Hz, suggesting a non-uniform cochlear response to treatment. Given the exploratory nature of this analysis and the limited sample size, mechanistic interpretation should remain cautious. These observations may also reflect measurement variability, test–retest differences, or other patient-related confounding factors. Nonetheless, the findings may indicate that conventional summary measures alone do not fully capture treatment-related auditory changes in AAV, supporting the complementary role of frequency-specific assessment.

All patients achieved complete (BVAS/WG = 0) or partial (BVAS/WG = 1–2) remission of the AAV after immunosuppressive treatment, with a mean baseline BVAS/WG score of 9.4 ± 4.2, confirming active systemic disease at inclusion. However, neither baseline disease activity nor the magnitude of BVAS/WG improvement correlated significantly with hearing outcomes. This dissociation suggests that systemic clinical remission does not necessarily equate to complete resolution of cochlear inflammation. The inner ear, characterized by limited regenerative capacity and specialized microvascular architecture, may exhibit delayed or incomplete functional recovery compared with other organs. Peripheral nervous system involvement was more frequently observed in patients with hearing loss. However, ABR testing did not reveal abnormalities in absolute latencies or interpeak intervals, arguing against retrocochlear or neural conduction pathology as the primary mechanism of sensorineural hearing loss in this cohort. These findings further support a predominantly cochlear rather than neural origin of auditory dysfunction in AAV.

No statistically significant differences in hearing outcomes were observed between rituximab and cyclophosphamide. Nonetheless, a higher proportion of slight improvement was noted in the rituximab group (9.7% vs. 5.3%). Given rituximab’s targeted B-cell depletion and its impact on ANCA production, it is conceivable that more precise immunomodulation may confer subtle advantages in microvascular stabilization. However, these observations remain exploratory and require validation in larger, prospective cohorts.

Otoacoustic emission (OAE) analysis did not demonstrate statistically significant improvement between visits (Table 4). Although DPOAEs and TEOAEs are sensitive indicators of outer hair cell integrity, their variability and susceptibility to noise may limit their utility in systemic inflammatory conditions. Prior reports have described reappearance of DPOAEs following remission [20]; however, in our cohort, OAE recovery was modest. In our previous study, we showed that OAEs may lack sufficient sensitivity for detecting early cochlear deterioration in AAV [19]. Nevertheless, DPOAEs—given their frequency specificity—may still provide complementary information during longitudinal monitoring, particularly when interpreted alongside HFA.

ABR findings were consistent with predominantly cochlear rather than retrocochlear sensorineural hearing loss. Mean wave V latency was 6.0 ± 0.4 ms (median 6.0 ms; range 5.4–7.1 ms) at baseline and 5.9 ± 0.4 ms (median 5.9 ms; range 5.2–7.2 ms) at follow-up, without evidence of clinically relevant conduction abnormalities.

Immittance audiometry findings suggest that auditory involvement in AAV is clinically heterogeneous and cannot be attributed exclusively to cochlear dysfunction (Table 5). Although tympanometric type A curves predominated in our cohort, a substantial subset of ears demonstrated conductive or mixed hearing loss, as well as abnormal tympanometric patterns consistent with middle ear dysfunction. These abnormalities were more frequent in patients with shorter disease duration, suggesting that middle ear involvement may be particularly relevant in earlier disease stages. Improvement in tympanometric findings during follow-up indicates that at least some conductive components may be dynamic and potentially reversible. This observation is consistent with the recognized spectrum of otologic manifestations in AAV, including Eustachian tube dysfunction, middle ear effusion, and otitis media associated with ANCA-associated vasculitis (OMAAV) [21,22]. No significant associations were found between disease duration and changes in pure-tone thresholds or OAEs, suggesting that cochlear dysfunction may occur independently of disease duration in at least a subset of patients (Table 5).

Collectively, our findings indicate that hearing impairment in AAV is most consistent with immune-mediated cochlear microvascular dysfunction, characterized by preferential high-frequency involvement and limited but potentially reversible recovery following induction therapy. These observations support the incorporation of high-frequency audiometry into routine monitoring protocols and highlight the need for early detection strategies aimed at preserving cochlear function in this patient population.

## 5. Study Limitations

This study has several limitations. First, the relatively small sample size reflects the rarity of ANCA-associated vasculitis and the prospective design, which may have limited the statistical power to detect significant associations between clinical and audiologic variables.

Second, the heterogeneity of the study population, including differences in disease phenotype, treatment regimens, and disease duration, may have contributed to variability in hearing outcomes. Although all patients were evaluated during active disease and achieved remission, the timing and extent of cochlear recovery may differ between individuals and could not be fully captured within the study timeframe.

Third, while a comprehensive audiologic test battery was applied, some measures—particularly otoacoustic emissions—may show limited sensitivity in systemic inflammatory conditions, potentially affecting the detection of subtle cochlear changes.

## 6. Conclusions

Monitoring hearing function and treatment outcomes constitutes a fundamental component of audiologic care, particularly in systemic inflammatory diseases with potential cochlear involvement. The use of established classification systems facilitates standardized outcome assessment and improves comparability across studies. In this context, grading systems developed for idiopathic sudden sensorineural hearing loss (SSNHL) may provide a valuable framework for evaluating hearing impairment in systemic conditions characterized predominantly by cochlear sensorineural pathology.

In the present study, we applied the Hearing Improvement Criteria for SSNHL defined by the Ministry of Health and Welfare of Japan to assess hearing severity and treatment-related changes in patients with ANCA-associated vasculitis (AAV). Despite the relatively small sample size and the limited number of statistically significant findings, our results suggest that this classification system may represent a practical and clinically meaningful tool for describing hearing abnormalities and monitoring therapeutic response in AAV.

We additionally explored potential associations between hearing outcomes and systemic disease parameters, including BVAS score, hearing loss grade, pattern of improvement, ANCA subtype, disease duration, and treatment modality. Most of these analyses did not demonstrate statistically significant relationships. This may reflect limited statistical power due to the modest cohort size rather than a true absence of association.

Overall, our findings support the incorporation of structured hearing outcome criteria and high-frequency audiometric assessment into the clinical monitoring of patients with AAV. Larger, prospective studies are warranted to validate these observations and to further elucidate the relationship between systemic disease activity and cochlear dysfunction.

## Figures and Tables

**Table 1:** Organ involvement according to hearing loss.

Organ/System Involvement	Hearing Status		
	Normal Hearing (*n* = 9)	Hearing Loss (*n* = 18)	*p*
eye			
Absent	8 (88.9%)	18 (100%)	0.333
Present	1 (11.1%)	0 (0%)	
CNS			
Absent	8 (88.9%)	15 (83.3%)	0.593
Present	1 (11.1%)	3 (16.7%)	
joints			
Absent	5 (55.6%)	10 (55.6%)	0.660
Present	4 (44.4%)	8 (44.4%)	
gastrointestinal tract			
Absent	9 (100%)	17 (94.4%)	0.667
Present	0 (0%)	1 (5.6%)	
heart			
Absent	9 (100%)	15 (83.3%)	0.279
Present	0 (0%)	3 (16.7%)	
peripheral nervous system			
Absent	8 (88.9%)	9 (50%)	0.057
Present	1 (11.1%)	9 (50%)	
kidneys			
Absent	2 (22.2%)	5 (27.8%)	0.571
Present	7 (77.8%)	13 (72.2%)	
skin			
Absent	6 (66.7%)	13 (72.2%)	0.774
Present	3 (33.3%)	5 (27.8%)	
paranasal sinuses			
Absent	5 (55.6%)	7 (38.9%)	0.340
Present	4 (44.4%)	11 (61.1%)	
lungs			
Absent	2 (22.2%)	4 (22.2%)	0.695
Present	7 (77.8%)	14 (77.8%)	

**Table 2:** Hearing loss improvement between first and second visit.

	Hearing Loss Grade			
Visit	G1 (*n* = Ears)	G2 (*n* = Ears)	G3 (*n* = Ears)	*p*
First	39 (78%)	6 (12%)	5 (10%)	0.52
Second	43 (86%)	3 (6%)	4 (8%)	

**Table 3:** Hearing loss improvement according to hearing loss grade.

Grade	Improvement Ears PTA 500–4000 [*n* = 50 Ears]				Improvement Ears PTA 9000–12,500 [*n* = 35 Ears]			
	No Change	Slight Improvement	Marked Improvement	Complete Recovery	No Change	Slight Improvement	Marked Improvement	Complete Recovery
I	38 (97.4%)	1 (2.6%)			36 (92.3%)	3 (7.7%)		
II	3 (50%)	3 (50%)			3 (50%)	3 (50%)		
III	5 (100%)	0(0%)			5 (100%)	0(0%)		
IV								

**Table 4:** Otoacoustic emissions improvement between first and second visits.

Visit	Overall TEOAE Response (*n* = Ears)	*p*	Overall DPOAE Response (*n* = Ears)	*p*
First	18 37.5%	0.555	23 50.0%	0.676
Second	20 43.5%		25 54.4%	

**Table 5:** Audiometric changes according to time to diagnosis of ANCA-associated vasculitis.

Audiometric Test Variable	Time to Diagnosis		
Pre-Treatment			*p*
	Group 1 (Disease Duration > 2 Months) (*n* = Ears)	Group 2 (Disease Duration < 2 Months) (*n* = Ears)	
Tympanometry B	4 (16.7%)	2 (10.5%)	0.001
Tympanometry C	1 (4.2%)	4 (21.1%)	
Tympanometry A	17 (70.8%)	8 (42.1%)	
Tympanometry As	1 (4.2%)	3 (15.8%)	
Tympanometry Ad	1 (4.2%)	2 (10.5%)	
OVERALL DPOAE response	14 (58.3%)	9 (40.9%)	0.238
OVERALL TEOAE response	11 (42.3%)	7 (31.8%)	0.330
PTA normal hearing	11 (42.3%)	11 (45.8%)	0.762
PTA sensorineural hearing loss	8 (30.8%)	4 (16.7%)	
PTA conductive hearing loss	3 (11.5%)	6 (25%)	
PTA mixed hearing loss	4 (15.4%)	3 (12.5%)	
**Post-treatment**			
Tympanometry B	3 (12.5%)	3 (15%)	0.002
Tympanometry C	0 (0%)	0 (0%)	
Tympanometry A	18 (75%)	9 (45%)	
Tympanometry As	2 (8.3%)	2 (10%)	
Tympanometry Ad	1 (4.2%)	6 (30%)	
OVERALL DPOAE response (*n* = ears)	14 (58.3%)	11 (50%)	0.571
OVERALL TEOAE response (*n* = ears)	14 (58.3%)	6 (27.3%)	0.034
PTA normal hearing	13 (50%)	11 (45.8%)	0.792
PTA sensorineural hearing loss	5 (19.2%)	6 (25%)	
PTA conductive hearing loss	1 (11.5%)	5 (20.8%)	
PTA mixed hearing loss	5 (19.3%)	2 (8.3%)	

## Data Availability

The data that supports the findings of this study is available upon request from the corresponding authors. The data is not publicly available due to privacy or ethical restrictions.

## Abbreviations

The following abbreviations are used in this manuscript (alphabetical order):

AAV	ANCA-associated vasculitis
ABRs	Auditory brainstem responses
ANCAs	Antineutrophil cytoplasmic antibodies
BVAS/WG	Birmingham Vasculitis Activity Score for Wegener’s Granulomatosis
CF	Cyclophosphamide
CG	Control group
CT	Computed tomography
CHL	Conductive hearing loss
DpOAE	Distortion product otoacoustic emissions
EGPA	Eosinophilic granulomatosis with polyangiitis
ENT	Ear, nose, throat
FEIAs	Fluorescent-enzyme immunoassays
GPA	Granulomatosis with polyangiitis
HFA	High-frequency pure-tone audiometry
MHL	Mixed hearing loss
MPA	Microscopic polyangiitis
(MPO)-ANCA	Myeloperoxidase ANCAs
OMAAV	Otitis media ANCA-associated vasculitis
(PR3)-ANCA	Proteinase 3 ANCAs
PTA	Pure-tone average
RTX	Rituximab
SG	Study group
SNHL	Sensorineural hearing loss
SSNHL	Sudden sensorineural hearing loss
SNR	Signal–noise ratio
SOAE	Spontaneous otoacoustic emissions
SPL	Sound pressure level
TEOAE	Transient-evoked otoacoustic emissions
